# Stem cell-derived extracellular vesicles: role in oncogenic processes, bioengineering potential, and technical challenges

**DOI:** 10.1186/s13287-019-1468-6

**Published:** 2019-11-26

**Authors:** Mujib Ullah, Yang Qiao, Waldo Concepcion, Avnesh S. Thakor

**Affiliations:** 1 Interventional Regenerative Medicine and Imaging Laboratory, Stanford University School of Medicine, Department of Radiology, 3155 Porter Dr., Stanford, CA 94304 USA; 2 Texas A&M University College of Medicine, 8447 Riverside Pkwy, Bryan, TX 77807 USA; 3 Department of Interventional Radiology, The University of Texas MD Anderson Cancer Center, 1515 Holcombe Blvd, Houston, TX 77030 USA; 4 Department of Surgery, Columbia University Irving Medical Center, 177 Fort Washington Ave, New York, NY 10032 USA

**Keywords:** Stem cells, Extracellular vesicles, Cancer, Inflammation, Immunology, Repair, Regeneration, Transplantation

## Abstract

Extracellular vesicles (EVs) are cellular-derived versatile transporters with a specialized property for trafficking a variety of cargo, including metabolites, growth factors, cytokines, proteins, lipids, and nucleic acids, throughout the microenvironment. EVs can act in a paracrine manner to facilitate communication between cells as well as modulate immune, inflammatory, regenerative, and remodeling processes. Of particular interest is the emerging association between EVs and stem cells, given their ability to integrate complex inputs for facilitating cellular migration to the sites of tissue injury. Additionally, stem cell-derived EVs can also act in an autocrine manner to influence stem cell proliferation, mobilization, differentiation, and self-renewal. Hence, it has been postulated that stem cells and EVs may work synergistically in the process of tissue repair and that dysregulation of EVs may cause a loss of homeostasis in the microenvironment leading to disease. By harnessing the property of EVs for delivery of small molecules, stem cell-derived EVs possess significant potential as a platform for developing bioengineering approaches for next-generation cancer therapies and targeted drug delivery methods. Although one of the main challenges of clinical cancer treatment remains a lack of specificity for the delivery of effective treatment options, EVs can be modified via genetic, biochemical, or synthetic methods for enhanced targeting ability of chemotherapeutic agents in promoting tumor regression. Here, we summarize recent research on the bioengineering potential of EV-based cancer therapies. A comprehensive understanding of EV modification may provide a novel strategy for cancer therapy and for the utilization of EVs in the targeting of oncogenic processes. Furthermore, innovative and emerging new technologies are shifting the paradigm and playing pivotal roles by continually expanding novel methods and materials for synthetic processes involved in the bioengineering of EVs for enhanced precision therapeutics.

## Background

**Fig. 1:**
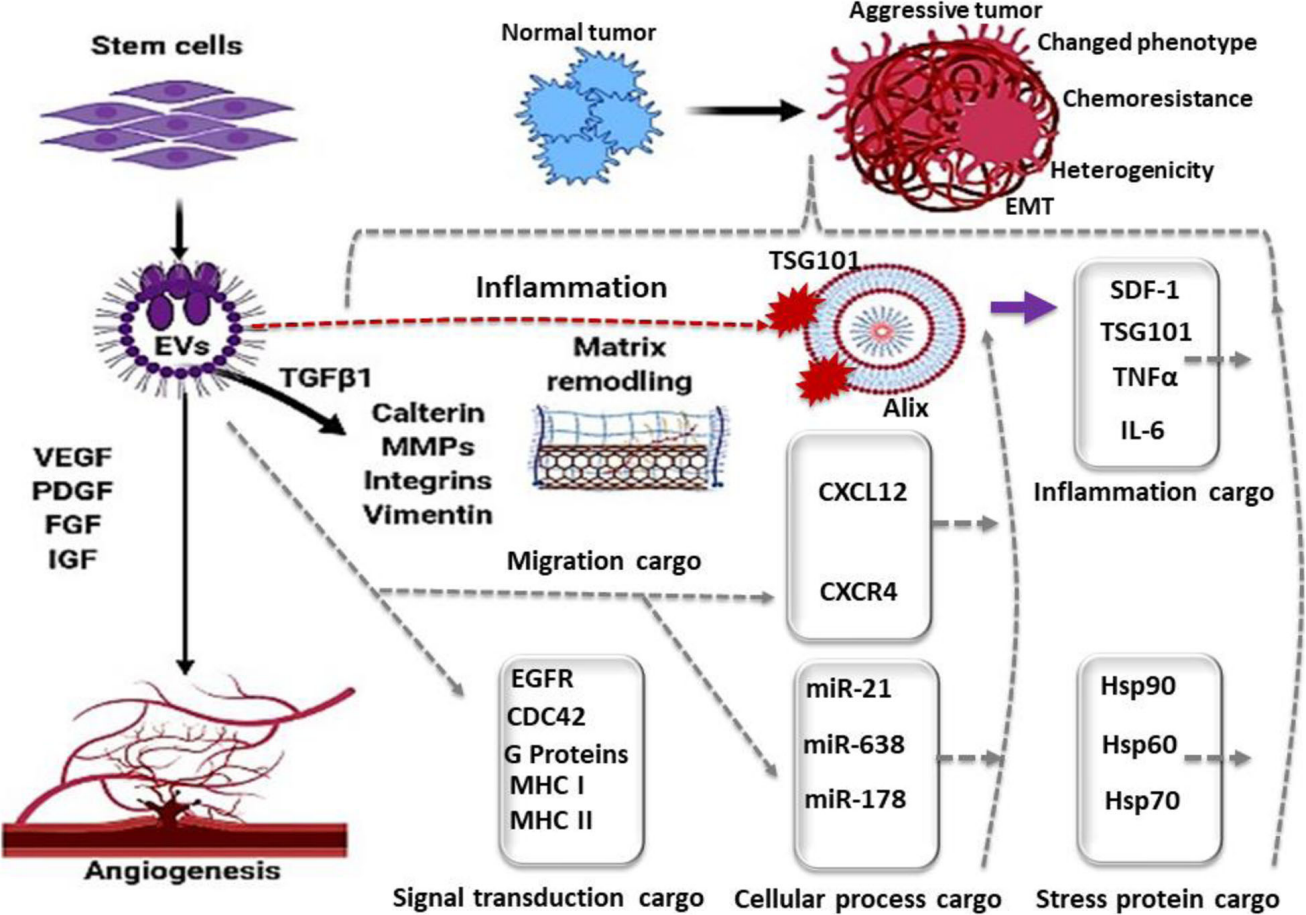
A schematic overview of the role of extracellular vesicles (EVs) in the cellular microenvironment. EV cargo loads may contain a diverse set of molecules such as growth factors (VEGF, PDGF, FGF), inflammatory mediators (Alix, TNFα, TSG, IL-6, SDF-1), heat shock proteins (HSP90, HSP70), and microRNAs (mir-21, mir-178)

Extracellular vesicles (EVs) have recently come to the attention of investigators as important biological entities with the unique capability for trafficking a variety of intercellular cargo, including lipids, proteins, and nucleic acids [1, 2], throughout the human body as well as in biological media such as blood, urine, breast milk, and cerebrospinal fluid [3]. Cells communicate with each other by exchanging information through the secretion of soluble factors such as growth agents, cytokines, and genetic material [4–7], all of which can be encapsulated within EVs [1]. EVs are also involved in the modulation of genetically encoded messages via miRNA trafficking and can program cells involved in repairing damaged tissue [8, 9]. Due to these pleiotropic effects, EVs are hypothesized to play an influential role in modulating the tissue microenvironment as it relates to the repair and regeneration of damaged or diseased tissues [3, 6, 10], as well as for the removal of unwanted proteins and toxic materials [6, 11]. These unique characteristics of EVs highlight their importance in understanding the pathophysiology of conditions as diverse as cancer, cardiovascular disease, infectious diseases, and neurodegenerative disorders [4, 6, 11–13] (Fig. 1).

**Fig. 2:**
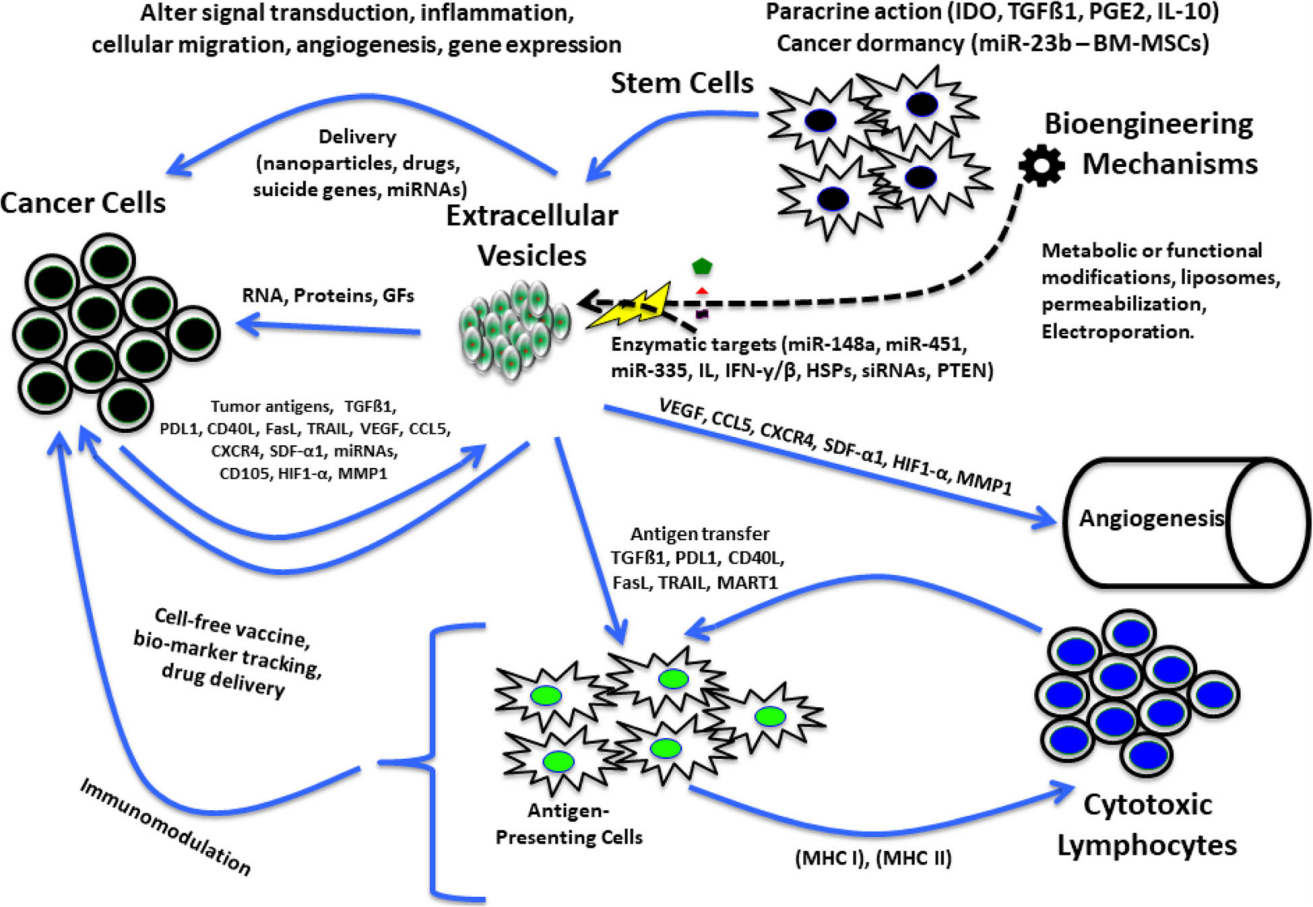
Schematic of interactions between EVs, stem cells, and cancer cells in the process of oncogenesis, and the differential bioengineering applications of EVs and stem cells for effecting anti-oncogenic activity

**Fig. 3:**
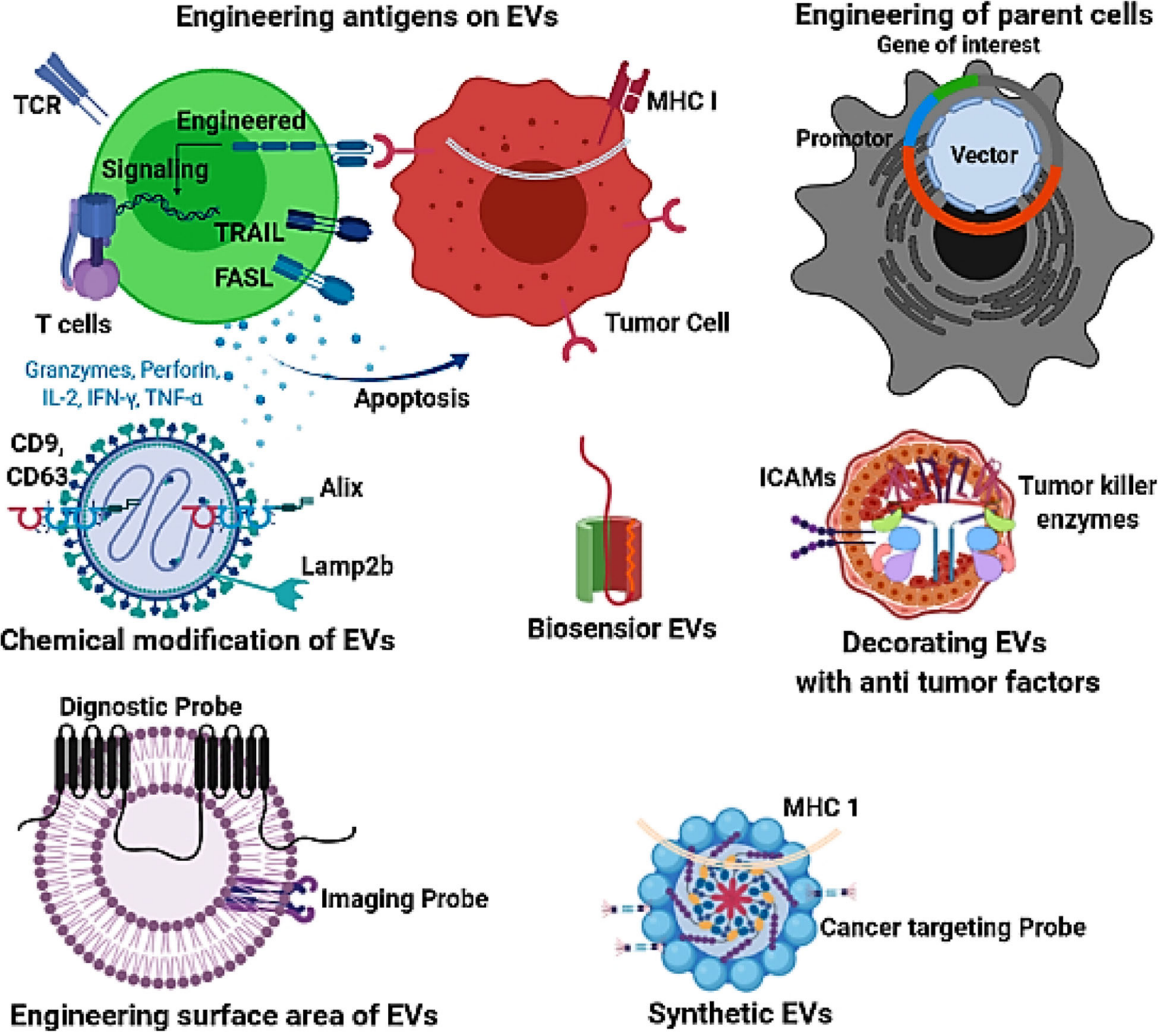
A schematic illustration demonstrating the diverse array of approaches available in EV engineering for enhancing effective therapeutic cargo and drug delivery in the treatment of cancer. These approaches include but are not limited to membrane surface antigen modifications, genetic modification of parental cells, chemical modification of EVs, sensor probe conjugation with EVs, conjugation of anti-tumor enzymes and proteins, and EV synthetic modifications

In the first half of this review, we will discuss the role of EVs in facilitating communication throughout the microenvironment, as well as their synergistic effects with bone marrow-derived stem cells in processes as diverse as inflammation, immunomodulation, cellular reprogramming, and tissue repair and regeneration (Fig. 1). In the second half of this review, we will dive into knowledge gathered from ongoing research concerning stem cell-derived EVs and evidence of their correlation with differing oncological states (Fig. 2). A greater understanding of the synergistic functions between EVs and stem cells is essential for a better understanding of cellular homeostasis and the pathogenesis of cancer and may also provide a novel delivery platform for new-generation therapies aimed at targeting inflammatory diseases and cancers through the development and utilization of bioengineered EVs (Figs. 2 and 3).

### Effects of stem cell-extracellular vesicle interactions on the cellular microenvironment

EVs play an important and fundamentally synergistic role with stem cells in various steps leading to the activation of immune and inflammatory pathways [4, 13–15]. Stem cell-derived EVs exert an immunomodulatory influence by reducing inflammation and enhancing the local immune response following the arrival of immune cells at the sites of tissue injury [2, 6, 13], including the modulation of both the primary and adaptive immune responses [13, 14]. Broadly, EVs derived from stem cells have been found to (i) promote survival of hematopoietic stem cells; (ii) activate monocytes, macrophages, and B cells [13, 14]; (iii) enhance the function of CD4+ regulatory T cells; and (iv) suppress the activity of natural killer and CD8^+^ cytotoxic T cells [13, 14] (Fig. 1).

EVs have also been reported to possess the capability for recruiting various types of stem cells scattered throughout the microenvironment to the sites of tissue injury and facilitate their coordination in tissue repair [8, 11, 16, 17]. Once mobilized stem cells reach the sites of tissue injury, they themselves can further secrete growth factors and cytokines in a paracrine manner to mediate the repair of damaged tissues and promote further wound healing [17–19]. Evidence suggests that stem cells migrate to the sites of tissue damage by following a chemo-attractant gradient and, once there, make connections with damaged cells to create replacement cells and complementary tertiary cells for tissue regeneration [1, 20–22]. Studies have shown stem cell-derived EVs to possess diverse capabilities including, but not limited to, educating bone marrow cells towards self-renewal [3, 23], regenerating skin following burn injury [2, 24, 25], inducing quiescent endothelial cells towards angiogenesis [2, 26–28], and influencing downstream phenotype differentiation of recruited stem cells.

Since the discovery of the immunomodulatory and regenerative properties of EVs, numerous animal models have demonstrated evidence of the important roles that stem cells and stem cell-derived EVs play in the treatment of a diverse array of diseases including, but not limited to, cardiac damage, arthritis, bone deformity, muscle degeneration, brain injuries, and cancer immunotherapy [12, 13, 29–31]. Of particular importance, numerous studies have shown the efficacy of stem cell-derived EVs for limiting infarct size and improving heart function following acute myocardial ischemic events as well as their efficaciousness in treating a variety of musculoskeletal insults as varied as bone and spinal disc injuries, and muscular degeneration [25, 32–35], highlighting the enormous potential for using stem cell-derived EVs for improving recovery following complex surgeries involving these tissues [25, 32–35] (Fig. 1).

Microenvironmental immunomodulatory effects are not only limited to EVs derived from mesenchymal stem cells but also involve those derived from stem cells coming from a diverse array of lineages. Recently, Oh and Jeon described the capability of transplanted neural stem cell-derived small EVs (NSC-sEVs) in reducing the extent of spinal cord injury via their effects on the microenvironment by decreasing neuronal apoptosis and neuroinflammation, while promoting autophagy of already-damaged tissues following the sentinel event, which led to an overall improvement in functional neuronal recovery [36]. EVs derived from induced pluripotent stem cells (iPSC) have also been shown to be isolatable for providing a therapeutic iPSC-based platform without incurring the normal risks associated with iPSC therapy such as the risk for tumorigenicity [37]. Additionally, by virtue of their pleiotropic signaling effects, EVs have also been shown to possess the capability of converting hematopoetic stem cells into liver cells, and bone marrow stem cells into lung cells [10, 23, 25].

Given their role in recruiting stem cells, modulating inflammatory responses, and catalyzing tissue repair, EVs represent a promising novel class of therapeutic agents that may be utilized by themselves for delivery of therapeutic cargo or as adjunctive agents for enhancing stem cell transplantation therapy at the sites of injury [11, 25].

### Extracellular vesicle and stem cell involvement in cancer growth and signaling

Tumors release various cytokines, growth factors, and inflammatory mediators that modulate immune responses in the surrounding tissue microenvironment [13, 15, 38]. These signals attract EVs and cells along a chemo-attractant gradient towards the site of the tumor [38, 39]. For example, directed migration and incorporation of EVs in metastatic tumors has been readily observed in tumors as varied as breast cancer, prostate cancer, and brain cancer, supporting the theory of EV-tumor tropism [38, 39]. The function of EVs in the tumor niche appears complicated and ambiguous due to contradictory results from different studies in different cancer cell lines. For example, increasing evidence has indicated that EVs exert both stimulatory and inhibitory effects in mice models of breast, ovarian, lung, and brain cancer [39, 40], suggesting that EVs can suppress or promote tumor growth and/or invasion depending on both their interactive behavior with microenvironments of different tumor subsets and the specific characteristics of their encapsulated cargo. Therefore, EVs may be involved in up- or downregulation of inflammation, immune surveillance, and metastatic checkpoint monitoring depending on the specific type or set of interactions between specific subsets of EVs and the specific tumor setting [13, 18] (Fig. 2).

Although EVs have been described to exhibit both stimulatory and inhibitory effects in a variety of cancer cell lines, they have mainly been described as a stimulator of tumor growth and progression due to their disruptive effects on the local inflammatory process and activity for challenging host immune surveillance activities [13, 38, 41–43]. Limited evidence have also suggested that EVs have stimulatory effects not only on cancer cell growth but also on tumor invasion and metastasis as well [39, 41, 43], which is the leading cause of death and mortality worldwide [43, 44]. Recent studies have described tumor microenvironmental modifications by EVs leading to the creation of an atmosphere more hospitable for progression of metastatic processes [18, 43, 44] with increasing evidence suggesting that EVs carry pathogenic proteins, growth factors, microRNA, and oncogenic factors that promote cancer spreading [39, 41, 43] (Fig. 2).

Although it is reported that cargo delivery by EV-derived apoptotic bodies to normal host cells leads to subsequent cell cycle arrest [39, 40, 45], cancer cells on the other hand have been described to utilize EVs for the delivery of pro-oncogenic factors to meet their ceaseless demand for nutrients and energy necessary for their rapid growth and continued survival [40, 45]. However, due to their lack of functional DNAse, Chk2, and defective p53 and p21 tumor suppressor proteins, cancer cells are conferred immunity against pro-apoptotic bodies [40, 45], while simultaneously highjacking the remaining functions of the EVs cargo delivery system for promoting their own growth and survival (Fig. 2). Due to these pro-oncogenic factors, a better and more detailed understanding of the tumor cell-EV interaction profile would help elucidate a more comprehensive understanding of cancer biology in the future and would provide a reasonable lead for developing a stem cell-based EV delivery platform for targeting cancer growth and metastasis.

### Bioengineered stem cell-derived EVs—a novel approach for cancer therapy

One important property of EVs is their strong tropism for migrating towards oncologic sites. This property makes them potentially valuable cellular vehicles for the delivery of therapeutic cargo involved in regulating a multitude of cancer signaling pathways [15, 39, 45]. Due to their oncotropic properties, several studies have identified EV-based therapies as a potential candidate platform amenable to bioengineering for targeting cancer growth and metastasis [38, 39, 43]. Administration of EVs transduced with anti-tumor therapeutic genes have already been described to decrease tumor growth size and limit metastasis in certain experimental models [18, 45], further supporting previously observed findings demonstrating the efficacy of local injection of genetically bioengineered EVs for suppressing metastasis in mice with resistant tumors [46, 47] (Figs. 2 and 3).

Positive oncolytic effects have also been demonstrated by EVs released from stem cells [39, 47]. Numerous studies have described stem cell-derived EVs loaded with exogenous anti-tumor enzymes, and then subsequently activated with a pro-drug, to successfully toxify and kill cancer cells [45–47]. Thus, stem cell-derived EVs have been proposed as an attractive transportation platform for the delivery of anti-tumor agents to especially difficult-to-reach locations, such as across the blood-brain barrier (BBB) for the treatment of intracerebral lesions [38, 44, 48]. In fact, bioengineered EVs have already been described to prevent brain tumor metastasis by trafficking anti-tumor proteins across the BBB towards remaining diseased portions of the brain parenchyma status post surgery of incompletely resected brain tumors [46, 49]. Similar studies using HCC1806, a more resistant and aggressive model of breast carcinoma in nude mice, also demonstrated a significant reduction in tumor burden following administration of bioengineered EVs [46, 49] (Fig. 2). Further investigation can thus reveal the potential for bioengineered stem cell-derived EVs to become a powerful transportation tool for treating cancer.

Despite the challenges and controversies surrounding stem cells, stem cell-derived EVs hold enormous potential as targets for cancer-related research and bioengineering in the development of oncologic treatment options. Genetically engineered EVs have already led to durable and reproduceable improvements in cancer therapy and drug delivery in select models [24, 44], with further advances poised to introduce new therapeutic proteins and epitope targets to the field [1, 24] (Fig. 2). Furthermore, the capability for in vitro maintenance of stem cell-derived EVs in the laboratory setting allows for the expedient screening of new EV-encapsulated drugs in determining their pharmacokinetic and pharmacodynamic properties prior to their translation into in vivo models. Further understanding of the interactions between cancer and stem cell-derived EVs remains a promising topic for developing a new generation of effective cancer treatment options centered around a platform involving stem cell-derived EV-based delivery therapies.

### Differential approaches for EV bioengineering in the development of advanced targeted cancer therapeutics

Engineered EVs have gained significant attention as a potential new platform in the development of a new generation of cancer immunotherapeutics [50, 51]. Strategies for EV modification are varied and can involve either modifications of parental cells or of EVs directly [51]. In the parental cellular modification approach, vectors can be used for inducing the expression of select proteins or noncoding RNA in progenitor cells [46, 52]. These modified parental cells can then package the newly expressed proteins into EVs for targeted export throughout the microenvironment [52]. Small interfering RNAs (siRNAs), drugs, and other small molecules can also be internalized into source cells in a similar manner and be released from parental cells via exocytosis [42, 46]. Alternatively, siRNAs, drugs, and small molecules can be targeted to EVs directly via genetic, metabolic, or surface molecular engineering methods [53, 54]. Direct EV modification can involve direct permeabilization of the EV membrane for the uptake of small molecules [52, 55] as well as surface modifications. EV surface modification can be achieved via biochemical means for a diverse array of molecules including, but not limited to, tumor-killing proteins and conjugation mediators, such as carbodiimides, which enable EV surface conjugation of chemotherapeutic molecules and specific siRNAs [53, 56] for targeted oncologic therapy (Fig. 3) (Table 1).

**Table 1:** Summary of the different approaches in the engineering of EVs for precision cancer therapeutics

Engineering approach	Examples	Advantages	Disadvantages	References
Overexpression of protein in parent cells	Rabies viral glycoprotein, CD63, GLUT4, HSPs, BDNF, CD24, EpCAM, CD3, SMPD2, HIF-1α	Enhanced cargo loading, efficient delivery, relatively simple, biocompatible, stable expression	Low transfection efficiency, contamination of non-transfected EVs, risk of genotoxicity	[51, 52, 57]
Antibody/antigen conjugation	CD9 antibody with Alexa-647, mCherry, photoreceptor cryptochrome 2, Nef-E7 fusion protein	Specific and easy to operate, targeted delivery, high therapeutic potential	May impair functionality, low loading efficiency, antigen immunogenicity	[50, 51, 55, 56]
Modification of surface proteins	Arg-Gly-Asp (RGD) peptide, Ac_4_ManNAz, PDGFR	Easy, effective for delivery, fast and scalable production, extended half life	Compromise membrane integrity, may change surface area	[24, 52, 55, 58]
Synthetic modification	EMMPRIN, MHC-I and MHC-II	Greater tracking efficiency, high drug loading efficiency	Toxicity, washing required, potential deformation of membrane	[51, 52, 54, 58, 59]
Chemical modification	Glypican-1, c(RGDyK) peptides	Enhanced fusion efficiency, better conjugation, stable binding	Toxicity, may impair functionality, harsh chemicals involved	[51, 54, 56, 59]
Passive and active loading (sonication, incubation, electroporation)	Paclitaxel, imatinib, siRNA, doxorubicin	Simple, intact membrane, quick and efficient, simple protocol, chemical free	Aggregation, slow passive loading, low efficiency, untargeted release of drugs	[51, 52, 54–57]

In addition to the traditional methods of EV bioengineering, the advent of new technologies is rapidly expanding the methods by which EVs can be synthesized and modified for targeted cancer therapies including the development of artificial EVs [51, 58]. Artificial EVs are semi-synthetic or fully synthetic EVs, which like native-bioengineered EVs, can also be conjugated via genetic engineering-based modifications to improve therapeutic efficacy against cancer [51, 55, 58]. One recent development in artificial EV-based therapy is the utilization of microfluidic-based technology, which provides an effective platform for antigenic modification, allowing for improved surface engineering of EVs to allow for enhanced imaging of cancer tissues and also enabling the production of intact MHC peptides to facilitate immunologically based tumor regression [51, 55, 58]. Studies utilizing microfluidic-based EVs decorated with melanoma tumor peptides (glycoprotein-100, MART-1, and MAGE-A3) on the EV surface revealed an enhanced ability for antigen presentation and T-cell activation [44, 54, 58] [54, 55, 58], with activation of glycoprotein-100-specific CD8+ T cells and effective reduction of melanoma tumor volume in mice [42, 54, 58]. Overall, antigen-specific CD8+ T-cell proliferation was significantly induced by artificially-engineered EVs compared to native, non-engineered EVs [54, 58, 60], supporting the idea that microfluidic-based technology has the potential to serve as an automated and highly-integrated platform for rapid real-time production of therapeutic EVs for the advancement of targeted cancer immunotherapy [55, 60] (Fig. 3) (Table 1).

Bioengineered EVs have demonstrated efficacy for the delivery of therapeutic molecules in a diverse array of oncological diseases, including but not limited to brain tumors, pancreatic cancer, colon carcinoma, breast cancer, and lung cancer [50, 54, 59]. This increased efficacy is due to the property of EV-tumor tropism, which allows for bioengineered EVs to be used for targeted delivery of therapeutic cargo towards cancerous cells [50, 54]. In general, bioengineered EVs divert cargo traffic away from non-cancerous tissues and towards cancerous tissues, and additionally enhance cellular uptake of diverted cargo [5, 42, 56]. Examples of the efficacy of this property have been demonstrated in multiple experiments including in a study involving rabies viral glycoprotein-modified EVs which were described to direct cargo traffic towards tumor tissues for the unloading of anti-tumor proteins [50, 51, 54]. Modified EVs expressing surface folate receptor-α were also described to preferentially-direct cargo towards brain cells, suggesting the potential for utilizing EVs for targeting brain tumors across the BBB [42, 48, 51]. In addition to surface-tagging, numerous studies have also demonstrated the anti-cancer efficacy of bioengineered EVs via intra-membranous loading with chemotherapeutic agents such as doxorubicin, 5-flurouracil, paclitaxel; or siRNA such as miR-134, anti-miR-503, miR-143 for reducing tumor growth [54, 56, 57, 59]. For example, intravenous injection of EVs loaded with doxorubicin via electroporation demonstrated a marked inhibitory effect on tumor cell growth [54, 56] (Fig. 3) (Table 1).

Apart from pure therapeutics, bioengineered EVs can also be used for diagnostic visualization of oncologic regions-of-interest, as demonstrated by in vivo tracking of EV-conjugated with near-infrared dye Xenolight DiR (1,1′-dioctadecyltetramethyl indotricarbocyanine iodide) following intravenous injection into tumor bearing mice [57] (Fig. 3) (Table 1).

It is well-known that EVs transport antigens loaded onto MHC class I and II complexes, and stimulate immune responses in relation to cancer prevention and homeostasis [50, 51, 58]. However, bioengineered EVs have a number of distinct advantages over native naïve EVs when it comes to cancer immunotherapy [54, 57, 58]. Bioengineered EVs can be designed to enhance the display of T-cell epitopes by inducing high-stability peptide-MHC-II complexes during targeted trafficking towards tumor regions [44, 50, 54, 58]. Enhanced tumor presentation of antigens by bioengineered EVs may stimulate T cells to attack tumors and lead to a correspondingly more aggressive anti-tumor immunologic response [42, 50]. Recently, several studies have described the enhanced immunologic phenomenon of bioengineered EVs in cancer therapy, demonstrating stronger CD8+ cytotoxic responses and anti-tumor immunity [42, 57] as well as promoting natural-killer cell activation and proliferation which was correlated to tumor regression [54, 56, 57]. Bioengineered EVs can also be pre-emptively loaded with viral or bacterial antigens for targeted activation of the immune system to fight against cancer. For example, dendritic cell-derived EVs loaded with viral antigens have demonstrated the capability for activating a CD8+ T-cell-mediated anti-tumor immunological response [43, 59, 61], while another study demonstrated the capability of bioengineered EVs conjugated to bacterial and viral antigens for activating macrophage and T-cell-mediated cytotoxic immune responses against cancer cells as well [57, 59, 60]. Additionally, unlike native EVs, bioengineered EVs would not contain any pro-oncologic growth factors that could be delivered to the tumor microenvironment due to the basis that they are engineered in a specific controlled environment.

In an alternative approach to bioengineering EVs for cancer immunotherapy, anti-tumor proteins can also be conjugated directly onto the membranes of EVs [54, 57]. For example, programmed death-ligand 1 (PD-L1) and tumor necrosis factor-related apoptosis-inducing ligand (TRAIL) engineered onto the surface of EVs demonstrated the ability to suppress tumor growth via CD8+ T-cell activation [51, 54, 61]. EVs can also be engineered to express microRNA Let-7a and GE11, a peptide that targets and binds to the epidermal growth factor receptor, which is displayed on a number of tumors of epithelial origin [50, 51, 56, 57]. Additionally, conjugation of CD147 onto the surface of EV membranes demonstrated a marked reduction of colorectal cancer in treated patients and presents as a promising potential EV-mediated immunotherapeutic cancer treatment option [51, 55, 56]. Similarly, EV membrane surfaces modified with EpCAM and CD24 demonstrate promising diagnostic and immunotherapeutic potential for ovarian cancer and deserve further future investigation as well [55, 57].

### Technical challenges

Despite the promising potential of bioengineered EVs, limitations to their development and utilization as therapeutic tools exist [51, 55]. These limitations include the possible precipitation of unexpected and undesired immune reactions, poor packaging efficiency of EVs, low stability, and high toxicity [54, 55, 58]. These challenges suggest a need for the development of artificial EVs that are more immunologically inert and with greater stability [50, 58]. However, since the synthetic design of engineered EVs depends wholly on their specific purpose of use, there is no one perfect protocol for EVs synthesis, and novel combinations of synthetic techniques could even possibly offer new insights and broaden the field of EV bioengineering [50]. Another technical challenge of bioengineered EVs is the fact that they are not well characterized due to their heterogenic nature, making it difficult to optimize dosages for human patient treatments [50, 51]. Additionally, many currently available EV bioengineering approaches, such as parental cellular transfection, suffer from poor yield, contamination, low purity, and high time-consuming operations [54, 57, 58].

Before bioengineered EVs can become a therapeutic reality, there is a need for improvements and standards within the currently available framework. Bioengineered EVs should be well characterized, a set of protocols should be developed and standardized, production of high-quality clinical grade EVs should be upscaled, and loading efficiency of therapeutic cargo should be improved. In order to facilitate the establishment of these necessary standards while making EVs therapy attainable for the public, there necessitates the availability of efficient and cost-effective technologies such as mass production of microfluidic systems in order to generate EVs with consistent characteristics and in sufficient quantities for clinical trials. Additionally, there is a need for greater understanding of the role of EVs in both pathology and in normal tissue, so that the risk of adverse and nosocomial side-effects can be properly assessed prior to the clinical administration of bioengineered EV therapy. The need for greater understanding is especially true regarding EVs crossing the blood-brain barrier, as there is very limited mechanistic knowledge about that specific application [50, 54]. Lastly, long-term safety and therapeutic effects of bioengineered EVs will be difficult to predict and should be assessed with long-term follow-up studies.

## Conclusions

EVs present a tremendous new opportunity in medicine for the development of a biologically based therapeutic platform for targeted drug, small molecular, and gene delivery [1, 11, 62]. Despite the enormous therapeutic potential of EV-based therapies, there is still much debate over the process of their purification, isolation, recovery, and interactive biology [5, 8]. Other open topics of discussion currently limiting the use of EVs in the clinical setting include (1) lack of an optimized EV production mechanism, (2) lack of an optimized visualizable platform for tracking and gauging EVs activity in vivo, (3) non-standardized purification and tagging protocols for the exploration of mechanistic release studies, (4) the undetermined native cargo of diverse arrays of EVs and their non-standardized potential for drug, small molecular, and gene loading, and (5) the undetermined exact mechanism of cargo delivery and incorporation into recipient cells. Further technical challenges include the determination of appropriate instruments and suitable cell culture media, standardization of methods for purification, recovery and characterization of purified EVs, and effective EV storage methods. Despite these challenges, however, EV engineering still presents as a tremendous opportunity for the development of an anti-oncologic platform. Due to the rapid advancement and integration of novel approaches in EV bioengineering, a more detailed understanding of the tumor-EV interaction profile would help elucidate a more comprehensive understanding of cancer biology overall and would provide both a promising and reasonable lead for developing a future stem cell-based EV delivery platform for targeting cancer proliferation and metastasis.

## Data Availability

Not applicable.

## Abbreviations

BBB: Blood-brain barrier
Chk2: Checkpoint kinase 2
EVs: Extracellular vesicles
p21: Cyclin-dependent kinase inhibitor 1 or CDK**-**interacting protein 1
P53: Tumor protein p53

## References

[1] De Jong OG, Van Balkom BW, Schiffelers RM, Bouten CV, Verhaar MC (2014). Extracellular vesicles: potential roles in regenerative medicine. Front Immunol.

[2] Buzas EI, Gyorgy B, Nagy G, Falus A, Gay S (2014). Emerging role of extracellular vesicles in inflammatory diseases. Nat Rev Rheumatol.

[3] Di Rocco G, Baldari S, Toietta G (2016). Towards therapeutic delivery of extracellular vesicles: strategies for in vivo tracking and biodistribution analysis. Stem Cells Int.

[4] Zhang B, Yeo RW, Tan KH, Lim SK (2016). Focus on extracellular vesicles: therapeutic potential of stem cell-derived extracellular vesicles. Int J Mol Sci.

[5] Tkach M, Thery C (2016). Communication by extracellular vesicles: where we are and where we need to go. Cell.

[6] Koniusz S, Andrzejewska A, Muraca M, Srivastava AK, Janowski M, Lukomska B (2016). Extracellular vesicles in physiology, pathology, and therapy of the immune and central nervous system, with focus on extracellular vesicles derived from mesenchymal stem cells as therapeutic tools. Front Cell Neurosci.

[7] Belov L, Matic KJ, Hallal S, Best OG, Mulligan SP, Christopherson RI (2016). Extensive surface protein profiles of extracellular vesicles from cancer cells may provide diagnostic signatures from blood samples. J Extracellular Vesicles.

[8] Riazifar M, Pone EJ, Lotvall J, Zhao W (2017). Stem cell extracellular vesicles: extended messages of regeneration. Annu Rev Pharmacol Toxicol.

[9] Ullah M, Stich S, Notter M, Eucker J, Sittinger M, Ringe J (2013). Transdifferentiation of mesenchymal stem cells-derived adipogenic-differentiated cells into osteogenic- or chondrogenic-differentiated cells proceeds via dedifferentiation and have a correlation with cell cycle arresting and driving genes. Differ.

[10] Haga H, Yan IK, Takahashi K, Matsuda A, Patel T (2017). Extracellular vesicles from bone marrow-derived mesenchymal stem cells improve survival from lethal hepatic failure in mice. Stem Cells Transl Med.

[11] Nawaz M, Fatima F, Vallabhaneni KC, Penfornis P, Valadi H, Ekstrom K, Kholia S, Whitt JD, Fernandes JD, Pochampally R (2016). Extracellular vesicles: evolving factors in stem cell biology. Stem Cells Int.

[12] Smith JA, Leonardi T, Huang B, Iraci N, Vega B, Pluchino S (2015). Extracellular vesicles and their synthetic analogues in aging and age-associated brain diseases. Biogerontology.

[13] Robbins PD, Morelli AE (2014). Regulation of immune responses by extracellular vesicles. Nat Rev Immunol.

[14] Holodick NE, Rothstein TL (2015). B cells in the aging immune system: time to consider B-1 cells. Ann N Y Acad Sci.

[15] Robbins PD, Dorronsoro A, Booker CN (2016). Regulation of chronic inflammatory and immune processes by extracellular vesicles. J Clin Invest.

[16] Phinney DG, Di Giuseppe M, Njah J, Sala E, Shiva S, St Croix CM, Stolz DB, Watkins SC, Di YP, Leikauf GD, et al. Mesenchymal stem cells use extracellular vesicles to outsource mitophagy and shuttle microRNAs. Nat Commun. 2015;6:8472. 10.1038/ncomms9472. 10.1038/ncomms9472 PMC4598952 26442449

[17] Ullah M, Sun Z (2018). Stem cells and anti-aging genes: double-edged sword-do the same job of life extension. Stem Cell Res Ther.

[18] Kanada M, Bachmann MH, Contag CH (2016). Signaling by extracellular vesicles advances cancer hallmarks. Trends Cancer.

[19] Stik G, Crequit S, Petit L, Durant J, Charbord P, Jaffredo T, Durand C (2017). Extracellular vesicles of stromal origin target and support hematopoietic stem and progenitor cells. J Cell Biol.

[20] Taverna Simona, Pucci Marzia, Alessandro Riccardo (2017). Extracellular vesicles: small bricks for tissue repair/regeneration. Annals of Translational Medicine.

[21] Teixeira JH, Silva AM, Almeida MI, Barbosa MA, Santos SG (2016). Circulating extracellular vesicles: their role in tissue repair and regeneration. Transfus Apher Sci.

[22] Ullah Mujib, Eucker Jan, Sittinger Michael, Ringe Jochen (2013). Mesenchymal stem cells and their chondrogenic differentiated and dedifferentiated progeny express chemokine receptor CCR9 and chemotactically migrate toward CCL25 or serum. Stem Cell Research & Therapy.

[23] Quesenberry PJ, Aliotta JM (2010). Cellular phenotype switching and microvesicles. Adv Drug Deliv Rev.

[24] Armstrong JPK, Holme MN, Stevens MM (2017). Re-engineering extracellular vesicles as smart nanoscale therapeutics. ACS Nano.

[25] Gyorgy B, Hung ME, Breakefield XO, Leonard JN (2015). Therapeutic applications of extracellular vesicles: clinical promise and open questions. Annu Rev Pharmacol Toxicol.

[26] Shigemoto-Kuroda T, Oh JY, Kim DK, Jeong HJ, Park SY, Lee HJ, Park JW, Kim TW, An SY, Prockop DJ (2017). MSC-derived extracellular vesicles attenuate immune responses in two autoimmune murine models: type 1 diabetes and uveoretinitis. Stem Cell Rep.

[27] Korbling M, Estrov Z (2003). Adult stem cells for tissue repair - a new therapeutic concept?. N Engl J Med.

[28] Johnson SM, Dempsey C, Chadwick A, Harrison S, Liu J, Di Y, McGinn OJ, Fiorillo M, Sotgia F, Lisanti MP (2016). Metabolic reprogramming of bone marrow stromal cells by leukemic extracellular vesicles in acute lymphoblastic leukemia. Blood.

[29] Ullah M, Sittinger M, Ringe J (2013). Extracellular matrix of adipogenically differentiated mesenchymal stem cells reveals a network of collagen filaments, mostly interwoven by hexagonal structural units. Matrix Biol.

[30] Helmke A, von Vietinghoff S (2016). Extracellular vesicles as mediators of vascular inflammation in kidney disease. World J Nephrol.

[31] Sun S, Chen G, Xu M, Qiao Y, Zheng S (2013). Differentiation and migration of bone marrow mesenchymal stem cells transplanted through the spleen in rats with portal hypertension. PLoS One.

[32] Morhayim J, Rudjito R, van Leeuwen JP, van Driel M (2016). Paracrine signaling by extracellular vesicles via osteoblasts. Curr Mol Biol Rep.

[33] Danielson KM, Das S. Extracellular vesicles in heart disease: excitement for the future? Exosomes Microvesicles. 2014;2(1). 10.5772/58390. 10.5772/58390 PMC4242103 25429310

[34] Gao T, Guo W, Chen M, Huang J, Yuan Z, Zhang Y, Wang M, Li P, Peng J, Wang A (2016). Extracellular vesicles and autophagy in osteoarthritis. Biomed Res Int.

[35] Oh SK, Jeon SR (2016). Current concept of stem cell therapy for spinal cord injury: a review. Korean J Neurotrauma.

[36] Rong Y, Liu W, Wang J, Fan J, Luo Y, Li L, Kong F, Chen J, Tang P, Cai W (2019). Neural stem cell-derived small extracellular vesicles attenuate apoptosis and neuroinflammation after traumatic spinal cord injury by activating autophagy. Cell Death Dis.

[37] Taheri B, Soleimani M, Fekri Aval S, Esmaeili E, Bazi Z, Zarghami N (2019). Induced pluripotent stem cell-derived extracellular vesicles: a novel approach for cell-free regenerative medicine. J Cell Physiol.

[38] D'Souza-Schorey C, Clancy JW (2012). Tumor-derived microvesicles: shedding light on novel microenvironment modulators and prospective cancer biomarkers. Genes Dev.

[39] Kosaka N, Yoshioka Y, Fujita Y, Ochiya T (2016). Versatile roles of extracellular vesicles in cancer. J Clin Invest.

[40] Hong IS, Lee HY, Kang KS (2014). Mesenchymal stem cells and cancer: friends or enemies?. Mutat Res.

[41] Rak J, Guha A (2012). Extracellular vesicles - vehicles that spread cancer genes. Bioessays.

[42] Wendler F, Favicchio R, Simon T, Alifrangis C, Stebbing J, Giamas G (2017). Extracellular vesicles swarm the cancer microenvironment: from tumor-stroma communication to drug intervention. Oncogene.

[43] Becker A, Thakur BK, Weiss JM, Kim HS, Peinado H, Lyden D (2016). Extracellular vesicles in cancer: cell-to-cell mediators of metastasis. Cancer Cell.

[44] Yokoi A, Yoshioka Y, Yamamoto Y, Ishikawa M, Ikeda SI, Kato T, Kiyono T, Takeshita F, Kajiyama H, Kikkawa F (2017). Malignant extracellular vesicles carrying MMP1 mRNA facilitate peritoneal dissemination in ovarian cancer. Nat Commun.

[45] Norozi F, Ahmadzadeh A, Shahrabi S, Vosoughi T, Saki N (2016). Mesenchymal stem cells as a double-edged sword in suppression or progression of solid tumor cells. Tumour Biol.

[46] Ullah M, Kuroda Y, Bartosh TJ, Liu F, Zhao Q, Gregory C, Reger R, Xu J, Lee RH, Prockop DJ (2017). iPS-derived MSCs from an expandable bank to deliver a prodrug-converting enzyme that limits growth and metastases of human breast cancers. Cell Death Discov.

[47] Ullah M, Akbar A, Ng NN, Concepcion W, Thakor AS (2019). Mesenchymal stem cells confer chemoresistance in breast cancer via a CD9 dependent mechanism. Oncotarget.

[48] D'Asti E, Chennakrishnaiah S, Lee TH, Rak J (2016). Extracellular vesicles in brain tumor progression. Cell Mol Neurobiol.

[49] Bartosh TJ, Ullah M, Zeitouni S, Beaver J, Prockop DJ (2016). Cancer cells enter dormancy after cannibalizing mesenchymal stem/stromal cells (MSCs). Proc Natl Acad Sci U S A.

[50] Yang M, Wu SY (2018). The advances and challenges in utilizing exosomes for delivering cancer therapeutics. Front Pharmacol.

[51] Liu C, Su C (2019). Design strategies and application progress of therapeutic exosomes. Theranostics.

[52] Yang B, Chen Y, Shi J (2019). Exosome biochemistry and advanced nanotechnology for next-generation theranostic platforms. Adv Mater.

[53] Nasiri Kenari A, Kastaniegaard K, Greening DW, Shambrook M, Stensballe A, Cheng L, Hill AF (2019). Proteomic and post-translational modification profiling of exosome-mimetic nanovesicles compared to exosomes. Proteomics.

[54] You B, Xu W, Zhang B (2018). Engineering exosomes: a new direction for anticancer treatment. Am J Cancer Res.

[55] Zhu Q, Heon M, Zhao Z, He M (2018). Microfluidic engineering of exosomes: editing cellular messages for precision therapeutics. Lab Chip.

[56] Luan X, Sansanaphongpricha K, Myers I, Chen HW, Yuan HB, Sun DX (2017). Engineering exosomes as refined biological nanoplatforms for drug delivery. Acta Pharmacol Sin.

[57] Gilligan KE, Dwyer RM. Engineering exosomes for cancer therapy. Int J Mol Sci. 2017;18(6). 10.3390/ijms18061122. 10.3390/ijms18061122 PMC5485946 28538671

[58] Garcia-Manrique P, Matos M, Gutierrez G, Pazos C, Blanco-Lopez MC (2018). Therapeutic biomaterials based on extracellular vesicles: classification of bio-engineering and mimetic preparation routes. J Extracellular Vesicles.

[59] Qiao Y, Gumin J, MacLellan CJ, Gao F, Bouchard R, Lang FF, Stafford RJ, Melancon MP (2018). Magnetic resonance and photoacoustic imaging of brain tumor mediated by mesenchymal stem cell labeled with multifunctional nanoparticle introduced via carotid artery injection. Nanotechnology.

[60] Yang Yoosoo, Hong Yeonsun, Cho Eunji, Kim Gi Beom, Kim In-San (2018). Extracellular vesicles as a platform for membrane-associated therapeutic protein delivery. Journal of Extracellular Vesicles.

[61] Zhao J, Vykoukal J, Abdelsalam M, Recio-Boiles A, Huang Q, Qiao Y, Singhana B, Wallace M, Avritscher R, Melancon MP (2014). Stem cell-mediated delivery of SPIO-loaded gold nanoparticles for the theranosis of liver injury and hepatocellular carcinoma. Nanotechnology.

[62] Ullah Mujib, Sun Zhongjie (2018). Klotho Deficiency Accelerates Stem Cells Aging by Impairing Telomerase Activity. The Journals of Gerontology: Series A.

